# Acute HIV Presenting With Unilateral Facial Nerve Paralysis: A Case Report

**DOI:** 10.7759/cureus.34062

**Published:** 2023-01-22

**Authors:** Omar Kandah, Shuo Liu, Peter Montesano

**Affiliations:** 1 Internal Medicine, Grand Strand Medical Center, Myrtle Beach, USA

**Keywords:** bell's palsy, facial nerve paralysis, hiv aids, hiv, bell’s palsy association

## Abstract

Cranial nerve VII palsy is one of the most common cranial nerve pathologies seen in clinical practice. In the vast majority of cases, the cause is thought to be idiopathic and is also referred to as Bell’s palsy. These cases are normally self-limiting and often treated with a short course of corticosteroids for symptom management. However, prompt work-up and diagnosis are crucial, as non-idiopathic causes can often be life-altering and necessitate prompt intervention. Here, we report a unique case of a 43-year-old immigrant male who presented to the emergency department with a three-day history of worsening facial droop and slurred speech, with associated facial pain, headaches, and dizziness for the previous week. On exam, there was stark right facial weakness involving both the upper and lower portions of the face with no sensory deficits. The patient’s right eye was erythematous and painful, with no ability to fully open or close the right eyelid. The initial workup showed minor transaminitis with pancytopenia. A thorough workup was initiated, and all testing and serology were normal, with the exception of initial HIV screening. This was then followed by polymerase chain reaction (PCR) and viral load testing, which confirmed a new diagnosis of acute HIV infection presenting with unilateral CN VII palsy. In this report, we discuss the etiology, clinical features, differentials, and treatment options for facial nerve paralysis, along with the subtle connection to acute HIV infection.

## Introduction

Facial nerve palsy is one of the most common cranial nerve pathologies seen in clinical practice. It is defined as acute ipsilateral paralysis of the facial nerve, resulting in weakness of the muscles of facial expression. This paralysis can be classified based on the level of neuronal involvement. Central facial nerve paralysis presents with paralysis of the lower part of the face only, with the lesion occurring on the opposite side of the weakness. In this process, the forehead muscles are spared. Peripheral facial nerve paralysis, however, presents with weakness on both the upper and lower portions of the face and is due to a lower-motor neuron dysfunction on the same side of the apparent weakness. Acute peripheral facial nerve palsy is often referred to as Bell’s palsy when the cause is thought to be idiopathic following extensive workup. Thus, Bell’s palsy is a diagnosis of exclusion. When causes are identified, it is commonly due to viral infection, most often Herpes simplex virus (HSV) [1]. However other viral causes have been shown to cause this clinical manifestation. Here, we report a case of unilateral facial nerve palsy that was found to be caused by acute HIV infection. 

## Case presentation

A 43-year-old immigrant male, with no past medical history, presented to the emergency department complaining of a three-day history of worsening facial droop, and slurred speech. He also noted mild facial pain, headaches, and progressive dizziness ongoing for the previous week. The patient described the pain as constant burning, radiating from his eye around the side of his head, along with pain all over his body. He also complained of a two-week history of epigastric pain as well. He denied any fevers, chills, recent illnesses, or sick contacts. He also denied recent coronavirus disease 2019 (COVID-19) exposure as well as receiving the vaccine. A previous history of chickenpox or shingles was also absent. He reported near-daily alcohol use of about one to two beers a day. Family history was non-contributory. He was also single and denied any sexual encounters though he was not forthcoming about it.

On exam, there was noticeable right facial weakness involving both the upper and lower parts of the face with no sensory deficits. The patient’s eye was significantly erythematous with no voluntary ability to open or shut the right eyelid. Minor gait unsteadiness was noted as well. Bulk muscle tone and strength in the upper and lower extremities were normal. There was some minor epigastric tenderness to palpation primarily located on the right upper quadrant. The remainder of the exam was essentially normal. Initial laboratory studies showed pancytopenia with elevated transaminases in the mid-100s.

**Figure 1:**
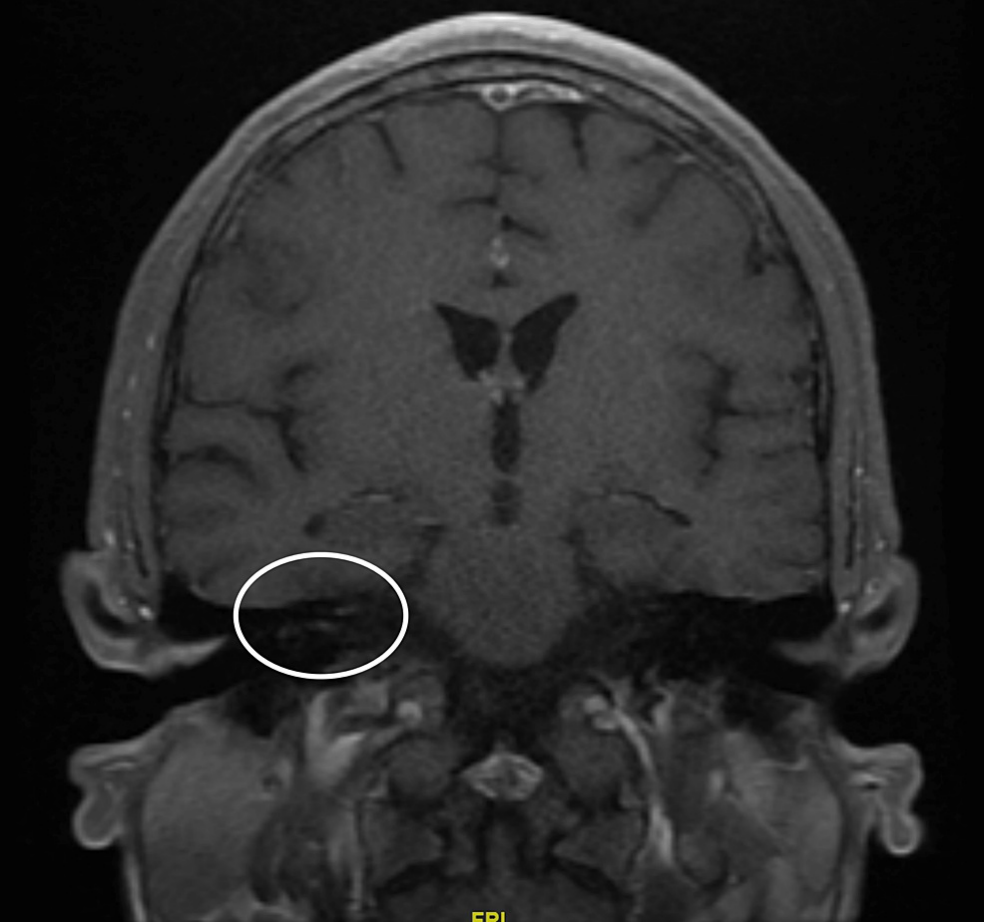
Brain MRI with and without contrast showing a punctate focus of enhancement in the distal right internal auditory canal

All diagnostic tests were essentially normal, including an entire stroke workup. Serology testing for major tick-borne illnesses, including Lyme and Rickettsii, as well as varicella zoster, viral hepatitis panel, HSV, and syphilis were all normal. An initial brain MRI was performed and was unremarkable. However, a repeat brain MRI was performed this time with and without contrast. This showed a punctate focus of enhancement in the distal right internal auditory canal, which could represent an abnormal enhancement of the right facial nerve, as can be seen in the setting of facial nerve palsy (Figure 1).

The only positive result to return to us was the HIV-1 antigen/antibody screen. Confirmatory tests proved to be positive as well, indicating an acute HIV-1 infection with viral RNA at the time of diagnosis, which resulted in 1,030,000 (PCR log10 6.013). Initial CD4 counts were depressed with an absolute CD4 count of 172. He was started on a prednisone taper and artificial tears. Infectious disease was consulted and made the decision to begin prophylactic trimethoprim-sulfamethoxazole daily, as well as bictegravir/emtricitabine/tenofovir alafenamide, one tab daily. He was then discharged following symptomatic improvement with the remainder of his steroid taper and was instructed to follow up with infectious disease as an outpatient, to manage his newly diagnosed HIV.

## Discussion

This case depicts peripheral facial nerve palsy as the initial presenting symptom of an underlying HIV-1 infection. Our patient originally presented to the emergency department with worsening facial droop and slurred speech, along with facial pain, headaches, and dizziness. Clinically, the patient appeared to be suffering from acute facial nerve palsy. Workup for acute cerebrovascular accident (CVA) along with the major viral and tick-borne illnesses was negative. Initial head imaging, including CT head and MRI of the brain, were unremarkable. It wasn’t until the patient had a repeat brain MRI with and without contrast that one was able to appreciate the abnormal enhancement of the right facial nerve. It was around this time that the result of HIV screening was positive. This was then followed by positive confirmatory testing of the HIV-1 virus, indicating that the patient was suffering from an underlying HIV-1 infection.

Bell’s palsy is defined as acute idiopathic facial nerve palsy, and it accounts for approximately half of all cases of peripheral facial nerve palsy [2]. Most recent data indicate the annual incidence rate to be about 13-34 cases per 100,000 population [3]. The majority of patients with idiopathic facial nerve palsy present with sudden onset facial paralysis, which includes both the upper and lower portions of the face. Symptoms tend to be unilateral but bilateral cases may occur as well. Bell’s palsy is a clinical diagnosis and can be made in patients with typical features, including unilateral facial weakness that involves both the lower and upper portions of the face with or without loss of taste of the anterior two-thirds of the tongue, which can occur acutely, within one to three days. While Bell’s palsy, by definition, is idiopathic, it is a diagnosis of exclusion. Other etiologies can, however, present with facial nerve palsy, including herpes simplex virus, herpes zoster, Lyme disease, otitis media, Guillain-Barre syndrome, and HIV infection, among others.

It is not uncommon for patients with primary HIV-1 patients to experience what is known as acute retroviral syndrome and current estimates indicate around 40-90% of patients do [4]. Patients suffering from acute retroviral syndrome typically exhibit fevers, myalgias, headaches, rashes, and lymphadenopathy soon after their primary exposure to HIV, as they begin the process of seroconversion. Rarely, patients may also develop neurological complications, including meningitis, encephalopathy, neuropathy, myelopathy, and neuritis [5]. Furthermore, in patients experiencing acute retroviral syndrome, Bell’s palsy has also been documented but mostly in patients who are already known to be infected with HIV [5]. There have been some case reports that demonstrate isolated facial nerve paralysis as the ﬁrst symptom of HIV-1 infection, occurring in association with acute infection or thereafter [6]. Furthermore, studies have shown that unilateral, along with bilateral, facial nerve paralysis is seen at a 100-fold greater frequency in patients with HIV-1 compared to general populations, (4.1% vs 0.04%) [7]. Interestingly, most associations with ipsilateral facial nerve paralysis and HIV infection are seen at the time of seroconversion, and many patients may initially have negative serologies at the time of presentation.

While this case demonstrates an example of Bell’s palsy due to HIV-1 infection, our patient’s serology during hospitalization showed a CD4 count of 172, indicating AIDS rather than acute onset or recent seroconversion [7]. Previous reports have focused on peripheral nerve palsy as a potential indication of new-onset HIV infection, rather than advanced disease [6]. Palsies seen later in the HIV course tend to be more likely caused by the secondary effects of lymphomas or other infectious etiologies. The patient described here showed no obvious signs of underlying malignancy or concomitant infection but a thorough workup of exclusion was not completed. The varied presentation in the onset of palsy in patients with HIV/AIDS showcases the relevance of our case, which depicted Bell’s palsy as the initial presenting symptom in late-stage HIV infection.

## Conclusions

Our case is a unique and interesting reminder that patients with both acute HIV infection, along with advanced HIV/AIDS disease, are still susceptible to common neurological disturbances such as facial nerve palsy. In patients who present to the hospital with the classic signs and symptoms of Bell’s palsy, it is important to keep HIV on the differential, in order to both explain the potential cause of the patient’s presenting facial paralysis, as well as set the patient up with appropriate HIV treatment and follow-up.
